# Reoperative transapical transcatheter aortic valve implantation for a degenerated biological valve

**DOI:** 10.1016/j.xjtc.2020.10.037

**Published:** 2020-11-03

**Authors:** Gabriella Ricciardi, Laura Cavallotti, Francesco Alamanni, Maurizio Roberto

**Affiliations:** a Department of Cardiothoracic Surgery, Leiden University Medical Center, Leiden, The Netherlands; b Department of Cardiac Surgery, Centro Cardiologico Monzino, Milan, Italy

**Figure:**
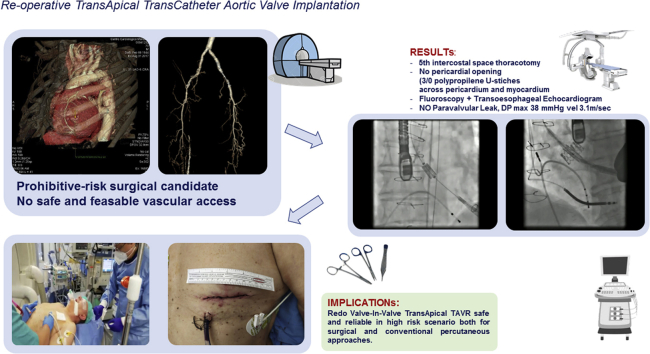

**Figure:**
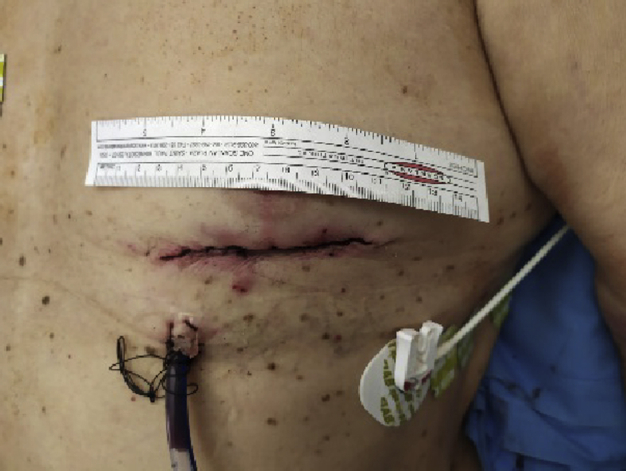
Left ventricular access used for the first transapical procedure on CT scan.

**Central Message**

Redo transapical aortic valve-in-valve is not recommended for device failure after primary transapical procedure, but it can be reliable in patients at risk for other transcatheter options or surgery.

See Commentary on page 121.

Transapical aortic valve implantation (TA-AVI) is a minimally invasive off-pump technique to treat aortic stenosis performed through a left anterolateral mini-thoracotomy exposing the apex of left ventricle (LV). The target population consists of elderly patients with high operative risk.

Redo TA valve-in-valve (VinV) for a degenerated prosthesis implanted during a primary TA- transcathether aortic valve implantation (TAVI) procedure is traditionally not recommended. The preferred approaches to this situation include a transfemoral or less common TAVI access or eventually a high-risk standard surgery. Going against this concept, we describe the case of a redo-TA aortic VinV implantation in presence of a degenerated transcatheter (TC) valve.

## Case Report

A 73-year-old patient with a degenerated aortic SAPIEN XT prosthesis (Edwards Lifesciences, Irvine, Calif) was referred to us. Of note in his history were coronary artery bypass graft, percutaneous coronary intervention, percutaneous transluminal angioplasty of iliac arteries and thromboendarterectomy of carotid arteries and femoral vessels, and severe chronic obstructive pulmonary disease. In 2010, he underwent a TA-AVI procedure with a 23-mm Edwards SAPIEN prosthesis. A pacemaker–implantable cardioverter-defibrillator was implanted 2 years later. The patient also reported several hospital admissions for heart and lung failure.

**Figure 1:**
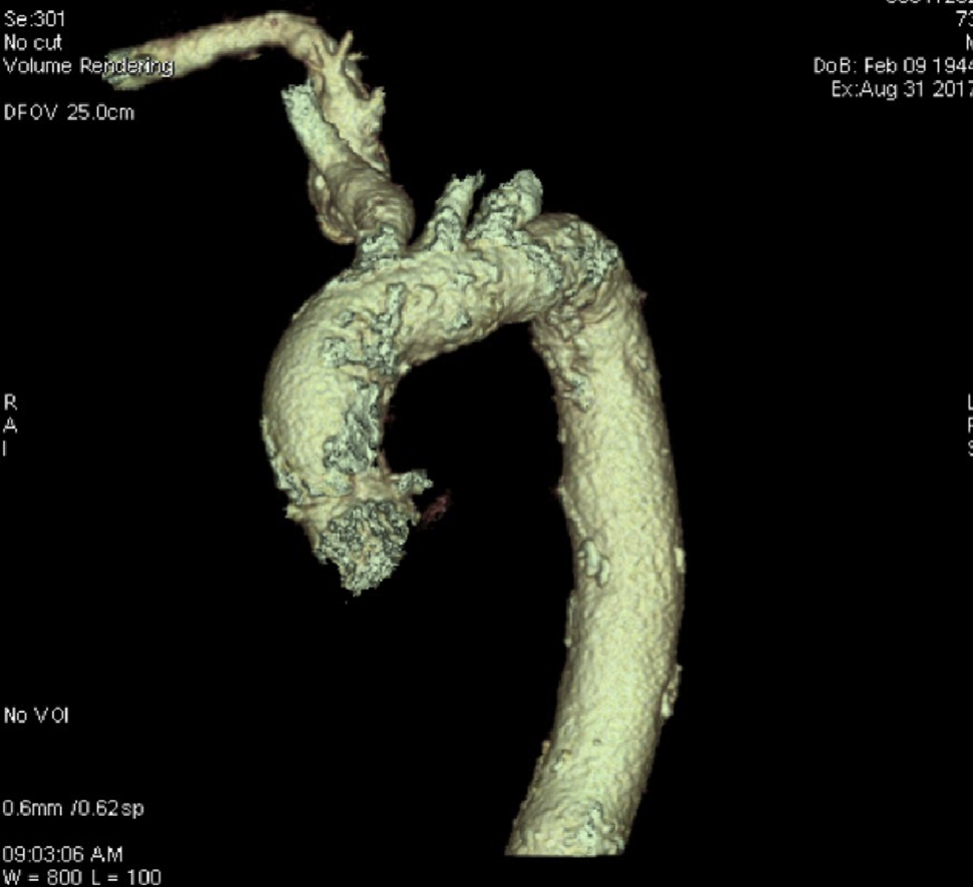
Computed tomography scan reconstruction showing the severe calcification of the ascending aorta, the arch, and head vessels.

**Figure 2:**
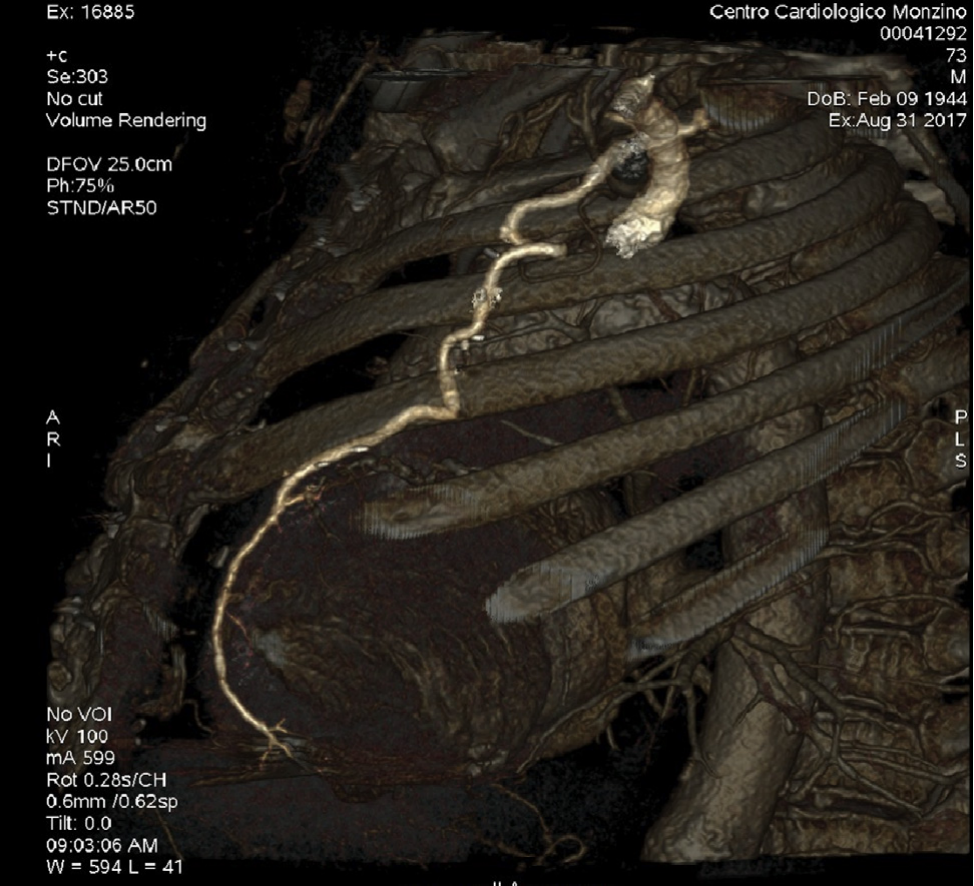
The history of the patient was notable for a previous coronary artery bypass grafting procedure. Computed tomography scan was performed to assess the patency of the anastomosis. In the figure, the left internal mammary artery to anterior descending coronary artery anastomosis is shown.

In 2017, he came to our emergency department with acute respiratory failure and pulmonary edema. A transthoracic echocardiogram (TTE) showed a degenerated aortic prosthesis leading to severe intra and paravalvular regurgitation. Computed tomography scan depicted the porcelain aorta (Figure 1), peripheral vasculopathy, patent coronary grafts (Figure 2), and marked the access site of the previous TC procedure.

**Figure 3:**
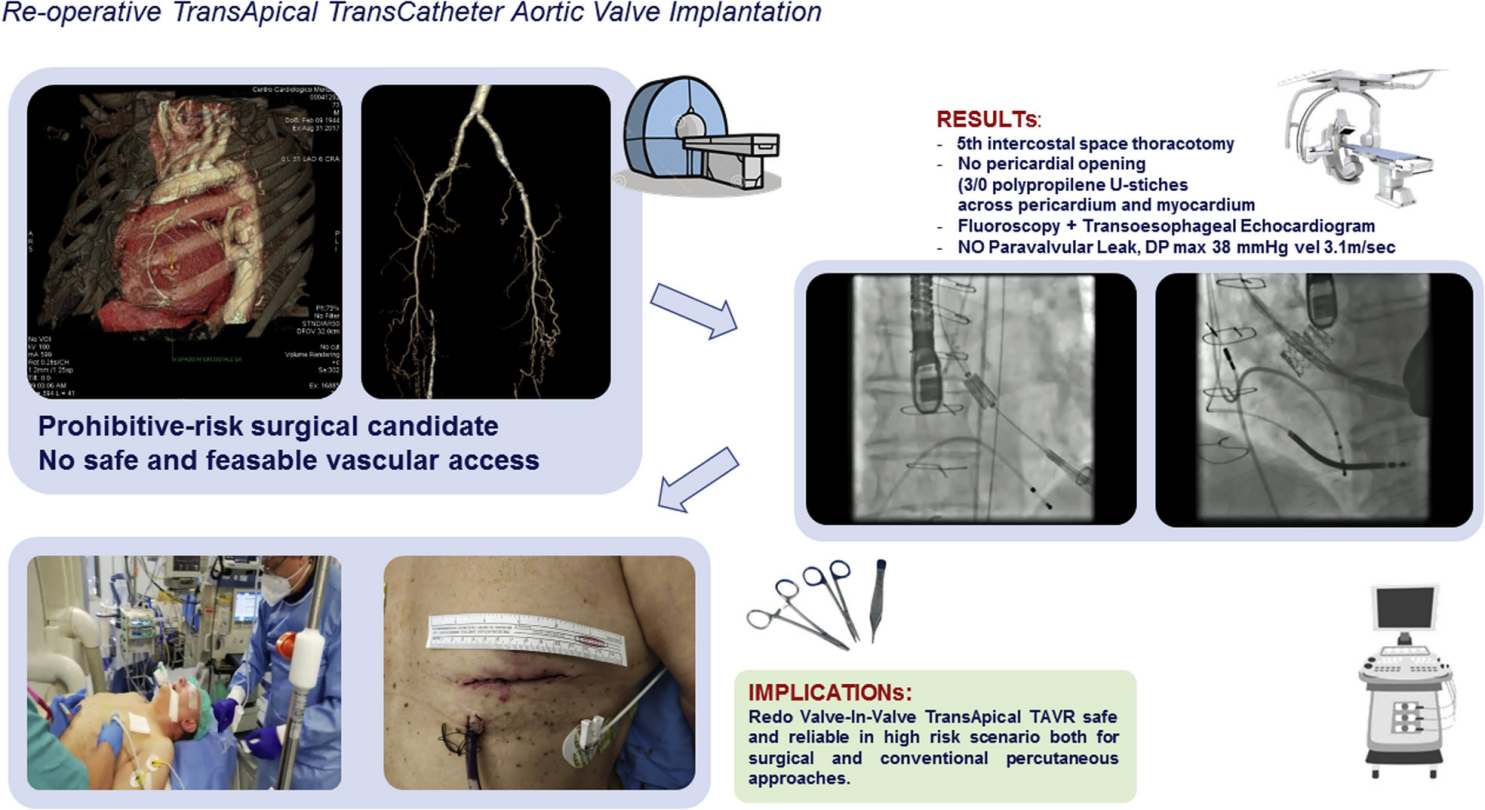
Brief description of the case and its management: computed tomography scan, catheter lab images, operating room setting, and skin wound at the end of the procedure.

The case management was discussed within the heart team. Concerns about patient's comorbidities (diffuse vasculopathy) and clinical history (several heart failure relapses) emerged. Therefore, it was judged more reliable to perform a VinV implantation through the previous TA access (TA redo TAVI) (Figure 3).

The procedure was conducted in the hybrid room through a re-thoracotomy at fifth intercostal space. A 6-F venous introducer was used in the left femoral vein. We avoided the pigtail catheter for the aortography considering the peripheral vasculopathy and the potential of fluoroscopy alone to ensure the proper view of the prosthesis' stent, which we used as a reference. The valve implantation was then performed under fluoroscopic and transesophageal echocardiographic guidance. A 7-F catheter was advanced into the LV apex, directly across the pericardium, and secured with two 3/0 PROLENE U-stitches, forming a Greek cross. The older prosthesis was crossed through a dedicate wire, switching then to the proper introducer (a 21-F Edwards Certitude introducer sheath) to implant a 23-mm SAPIEN 3 (Edwards Lifesciences) bioprosthesis into the older one, under 180 beats-ventricular pacing. The introducer was removed and the left apical stitches carefully ligated, again during ventricular pacing. No complications occurred.

The postprocedural TEE measured a maximal gradient of 38 mm Hg and a mean velocity of 3.1 m/s. The postoperative course was uneventful. The TTE confirmed the regular function and position of the valve, without any paravalvular leak. The patient was discharged on the ninth postoperative day. At 1 year, the TTE confirmed a good result, with a mean gradient across the valve of 28 mm Hg and preserved LV function (ejection fraction 70%) (Video 1).

**Video 1:**
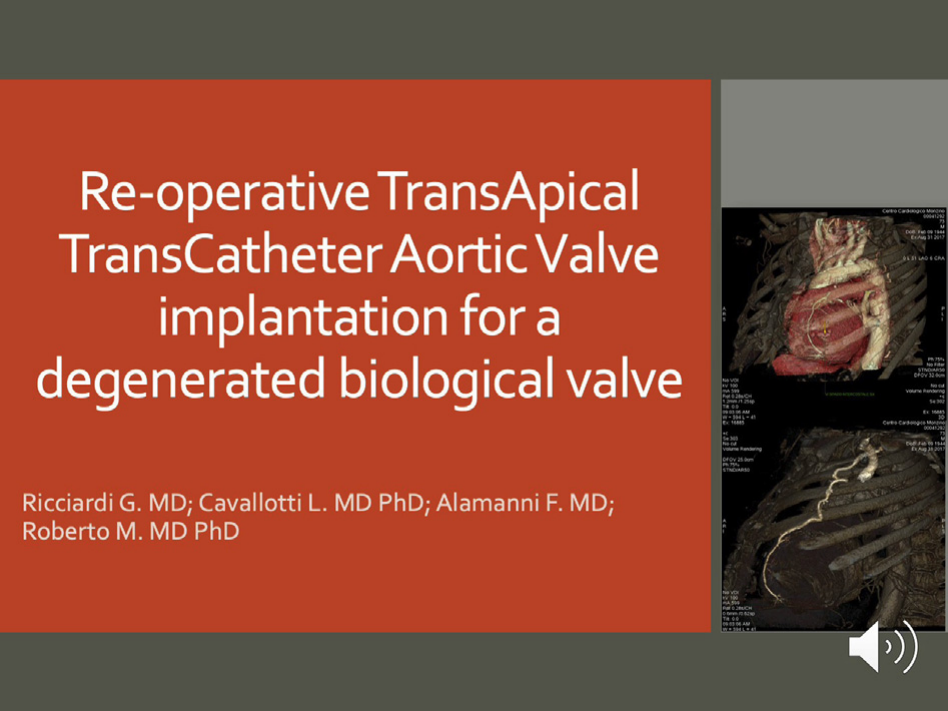
We performed a cardiac CT scan in our patient to demonstrate the presence of porcelain aorta, severe peripheral vasculopathy, the patency of the grafts, and finally to identify properly the site of the sheath introduction across the left ventricular apex. The heart team discussion confirmed the indication for a redo transapical valve in valve aortic implantation. In the video, we show our hybrid operating room and transthoracic preprocedural echocardiogram to identify the left ventricular apex and then make a short and precise incision at the fifth intercostal space. Because it was a redo case, we directly crossed the pericardium without opening it with a 7-F catheter, followed by a 21-F sheath. Two 3/o polipropylene U stitches, forming a Greek cross, were passed through both pericardium and myocardium of the left ventricle. In the bottom, on the left side, a short video of the VinV procedure carried under fluoroscopic guide is shown: the valve opening was performed very quickly during a rapid ventricular pacing. In the bottom, on the right side, you can see the same opening procedure under transesophageal echocardiographic guide. We then show the transesophageal echocardiographic check a few minutes after valve implantation, which showed the absence of paravalvular leaks and a maximal gradient of 38 mm Hg across the prosthesis, with a velocity of 3.1 m/s. The postoperative course was uneventful, and the patient was discharged on the ninth postoperative day. Video available at: https://www.jtcvs.org/article/S2666-2507(20)30625-8/fulltext.

Permission was granted by the patient's respective parents to publish the proposed case report.

## Comment

Only 2 reports exist on the possible use of TA-AVI as an effective treatment option in a failed TC valve.^1^^,^^2^ Less is known about the results and the feasibility of a VinV TA approach to address a degenerated TA-TC valve.^3^^,^^4^

In the presented case, the discussion within the heart team pointed out concerns regarding the patient's management: the diffuse, severe vasculopathy contraindicated a transfemoral TAVI, whereas respiratory problems and graft patency posed conventional surgery at an unreasonable risk. Conversely, repeating the TA access showed potential benefits. First, opting for a transpericardial approach to eliminate risks carried by adhesiolysis to reach the ventricle, the procedure was judged adequately safe. Likewise, the antegrade access, the orthogonal positioning, the defined landing zone with the previous stent, combined with porcelain aorta, severe peripheral arteriopathy, further favored the TA option over transvascular retrograde AVI, as in the case described by Kiefer and colleagues.^4^

In a prospective study by Kempfert and colleagues,^5^ the technical feasibility of TA VinV implantation in patients affected by different types of degenerated aortic xenografts has been already analyzed, showing excellent outcomes.

According to our single experience, we believe that it is of worth to further investigate the supposed good results of the TA approach in frail patients with a failed TC valve approaching a larger cohort by a randomized controlled trial or at least an observational study. Such studies could ease the broadening of the procedure as well as the expansion of its current indication in this setting.

## Supplementary Data

Video 1 We performed a cardiac CT scan in our patient to demonstrate the presence of porcelain aorta, severe peripheral vasculopathy, the patency of the grafts, and finally to identify properly the site of the sheath introduction across the left ventricular apex. The heart team discussion confirmed the indication for a redo transapical valve in valve aortic implantation. In the video, we show our hybrid operating room and transthoracic preprocedural echocardiogram to identify the left ventricular apex and then make a short and precise incision at the fifth intercostal space. Because it was a redo case, we directly crossed the pericardium without opening it with a 7-F catheter, followed by a 21-F sheath. Two 3/o polipropylene U stitches, forming a Greek cross, were passed through both pericardium and myocardium of the left ventricle. In the bottom, on the left side, a short video of the VinV procedure carried under fluoroscopic guide is shown: the valve opening was performed very quickly during a rapid ventricular pacing. In the bottom, on the right side, you can see the same opening procedure under transesophageal echocardiographic guide. We then show the transesophageal echocardiographic check a few minutes after valve implantation, which showed the absence of paravalvular leaks and a maximal gradient of 38 mm Hg across the prosthesis, with a velocity of 3.1 m/s. The postoperative course was uneventful, and the patient was discharged on the ninth postoperative day. Video available at: https://www.jtcvs.org/article/S2666-2507(20)30625-8/fulltext.
